# The genus *Dixa* (Diptera, Dixidae) in Croatian lotic habitats, with a checklist of species and relationships with the fauna of neighbouring countries

**DOI:** 10.3897/zookeys.867.36613

**Published:** 2019-07-29

**Authors:** Marija Ivković, Lara Ivanković

**Affiliations:** 1 Division of Zoology, Department of Biology, Faculty of Science, University of Zagreb, Rooseveltov trg 6, 10000 Zagreb, Croatia University of Zagreb Zagreb Croatia; 2 Zeleni trg 2, 10000 Zagreb, Croatia Unaffiliated Zagreb Croatia

**Keywords:** Meniscus midges, aquatic insects, faunistics, ecology

## Abstract

Invertebrate surveys in Croatia conducted between 2005 and 2018 included 39 sampling sites yielding bycatch samples of Dixidae (Diptera). All records of this family from the territory of Croatia are summarized, including previously unpublished data. Collections contained six species of *Dixa* Meigen – *D. dilatata* Strobl, *D. maculata* Meigen, *D. nebulosa* Meigen, *D. nubilipennis* Curtis, *D. puberula* Loew, and *D. submaculata* Edwards, with *Dixa dilatata* reported from Croatia for the first time. Information relating to the ecoregions in which species were found and specific species traits are provided. Compared to neighbouring countries, the Croatian species assemblage is most similar to the fauna of Italy and least similar to that of Serbia and Montenegro.

## Introduction

The Dixidae, or meniscus midges, are one of the smallest families of Diptera in Europe, with only two genera, *Dixa* Meigen and *Dixella* Dyar and Shannon, and 32 species recorded (Oosterbroek 2007; Pape and Beuk 2012). Approximately 190 species are recognized worldwide (Wagner et al. 2008; Pape et al. 2011; Moulton 2016, 2017). They are nematocerous flies belonging to the superfamily Culicoidea, which also includes the Culicidae, which they most closely resemble (Wiegmann et al. 2011; Borkent 2012). Adults are small, frail, and do not feed. They remain near their biotopes (streams, ponds), and rest in the vegetation. Males of some species form swarms. Eggs are deposited in masses at the water’s edge, and the life cycle includes four larval instars and the pupa. The larvae are filter feeders that rest on the water surface where they take on a characteristic, reversed U-shaped posture. Pupation takes place on emergent substrates. Larvae are feeding on microorganisms and decaying plant or animal material trapped in the water column or on the surface film (Wagner et al. 2008, Wagner 2011). Larvae of *Dixa* prefer running water, while those of *Dixella* occur in both stagnant or slow-moving water (Oosterbroek 2007). Some species are restricted to bog or mesotrophic lakes and are appropriate bioindicators. Species diversity is highest in springs and in headwater streams (Wagner et al. 2008). Dixid larvae are sometimes a significant component of invertebrate drift in streams (Elliott and Tullett 1977; Sertić Perić et al. 2014). They are extremely sensitive indicators of the presence of surfactant or oil-borne pollutants in streams (Thomas 1979). Larval mortality increases with decreasing surface tension of water (Fowler et al. 1997). Disney (1999) published an exceptionally fine compilation of West Palaearctic Dixidae that can be used worldwide as a basic information resource.

So far there have only been two studies dealing with Dixidae in Croatia, and the only records are those in Ivković et al. (2017) from the Krka River and Ivanković et al. (2019) from Plitvice Lakes National Park resulting from a study of the emergence patterns and ecological preferences of Dixidae.

## Materials and methods

**Study site.** Croatia is a relatively small country with a surface area of 56,594 km^2^ situated at the crossroads of Central and Mediterranean Europe and the Balkan Region. It is divided into two ecoregions, the Dinaric western Balkan (ER5) and the Pannonian lowland (ER11) (Illies 1978), and forms part of two drainage basins, the Black Sea Basin and the Adriatic Sea Basin.

**Figure 1.**
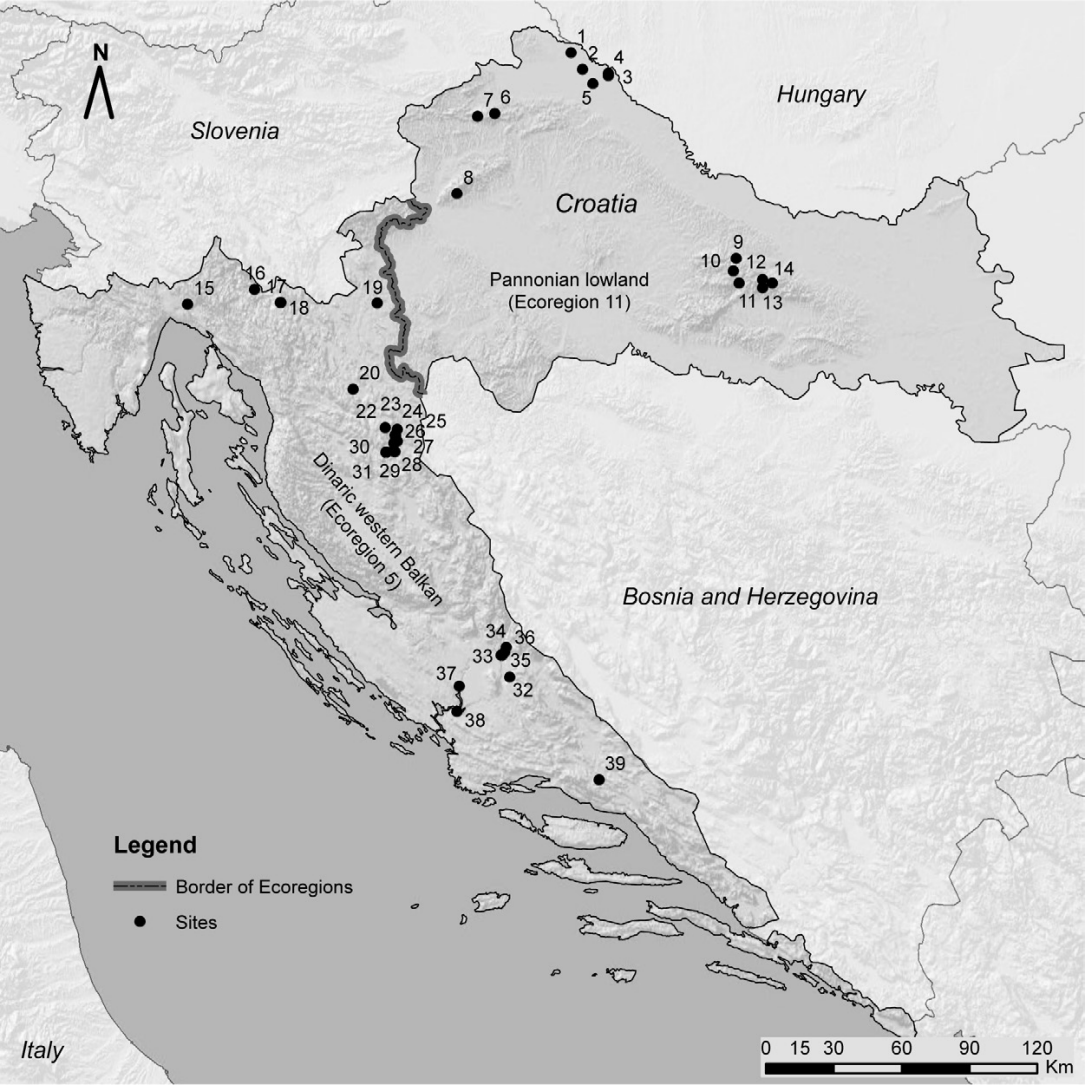
Sampling sites of *Dixa* recorded from Croatia (see Table 1 for codes).

**Specimen records.** This paper is based on unpublished data from our own research and published data gleaned from the literature. Each record was georeferenced using ArcGIS software. The literature used for identifications included Shtakel’berg (1989) and Disney (1999). We followed the current classification of Pape and Beuk (2012). Locality records are listed for each species. A list of locality names including latitude, longitude, altitude, and number code for each locality is given in Table 1, and a map with all sites plotted is provided as Figure 1. Specimens were collected from lotic freshwater habitats throughout Croatia. Adult specimens were collected using emergence traps (details in Ivković et al. 2013), sweep nets, yellow pan traps and aspirators, whereas larvae were collected by Surber sampler (25 × 25 cm) and kick-net sampler (25 × 25 cm). Larval samples were collected as a result of several macroinvertebrate surveys conducted between 2005 and 2018. Specimens were preserved in 80% or 96% ethanol (EtOH). For identification of adults, male and female terminalia were dissected, if needed. In some cases, terminalia (and preceding abdominal segments) were cleared in 10% KOH, neutralized with acetic acid, and rinsed in water to improve visualization and facilitate identification. For larvae, all available structural characters were used for identification. Taxonomic diversity was considered at the level of species according to Pape and Beuk (2012). European ecoregions were defined according to the *Limnofauna Europaea* (Illies 1978).

**Table 1.** Sampling sites in Croatia. Ecoregions are taken from Illies (1978); Dinaric western Balkan (5) and Pannonian lowland (11).

**Site Name**	**Site ID**	**Latitude**	**Longitude**	**Elevation (m)**	**Ecoregion**
Bošćak Stream	1	46°25'45"N, 16°35'48"E		145	11
Bistrec Stream, Rakovnica	2	46°21'50"N, 16°39'43"E		145	11
Bistrec Stream	3	46°20'17"N, 16°48'34"E		145	11
Kotoribski kanal	4	46°20'53"N, 16°48'41"E		130	11
Plitvica, Upper Reach	5	46°18'20"N, 16°43'21"E		205	11
Spring Šumi, Zagorje	6	46°11'19"N, 16°09'27"E		390	11
River Reka, upper reach	7	46°10'33"N, 16°03'38"E		170	11
Kraljevec, Medvednica Mountain	8	45°52'03"N, 15°56'45"E		420	11
Djedovica by Rupnica, Papuk Mountain	9	45°36'17"N, 17°31'54"E		365	11
Brzaja, before N. Zvečeva, Papuk Mountain	10	45°33'17"N, 17°30'53"E		500	11
Stream Kovačica, Papuk Mountain	11	45°31'12"N, 17°40'51"E		360	11
River Duboka rijeka, Papuk Mountain	12	45°30'26"N, 17°32'53"E		355	11
Headwater of Dubočanka, Papuk Mountain	13	45°30'17"N, 17°44'03"E		670	11
Dubočanka, Papuk Mountain	14	45°29'11"N, 17°40'42"E		585	11
River Riječina, upper reach	15	45°24'30"N, 14°25'30"E		380	5
Velika Belica	16	45°28'27"N, 14°48'12"E		430	5
Headwater of Dobra River	17	45°25'28"N, 14°57'04"E		539	5
River Bukovačka Dobra	18	45°25'23"N, 14°57'15"E		515	5
Mrežnički Brig, Mrežnica River	19	45°25'34"N, 15°29'51"E		130	5
River Dretulja, Plaški	20	45°05'03"N, 15°22'09"E		370	5
River Korana in Korana village, NP Plitvice	21	44°55'33"N, 15°37'09"E		390	5
Stream Sartuk, NP Plitvice	22	44°55'57"N, 15°33'10"E		765	5
Stream Plitvica, NP Plitvice	23	44°54'08"N, 15°36'27"E		555	5
Tufa barrier Novakovića Brod, NP Plitvice	24	44°54'08"N, 15°36'38"E		505	5
Tufa barrier Kozjak-Milanovac, NP Plitvice	25	44°53'39"N, 15°36'32"E		545	5
Lake Kozjak, NP Plitvice	26	44°52'40"N, 15°37'07"E		535	5
Tufa barrier Labudovac, NP Plitvice	27	44°52'17"N, 15°35'59"E		630	5
Middle reach of Crna rijeka, NP Plitvice	28	44°50'22"N, 15°35'59"E		665	5
Upper reach of Crna rijeka, NP Plitvice	29	44°50'10"N, 15°36'30"E		670	5
Upper reach of Bijela rijeka, NP Plitvice	30	44°50'04"N, 15°33'33"E		715	5
Spring of Bijela rijeka, NP Plitvice	31	44°49'58"N, 15°33'25"E		720	5
Kosovčica Spring	32	43°56'27"N, 16°15'09"E		255	5
River Kosovčica	33	44°01'39"N, 16°12'45"E		220	5
River Orašnica	34	44°03'40"N, 16°13'59"E		225	5
River Krka above the mouth of River Kosovčica	35	44°02'24"N, 16°13'27"E		215	5
River Krka below the mouth of River Kosovčica	36	44°01'40"N, 16°12'19"E		210	5
Roški Slap, Krka River, NP Krka	37	43°54'13"N, 15°58'29"E		55	5
Skradinski buk, Krka River, NP Krka	38	43°48'14"N, 15°57'52"E		25	5
Čikotina Lađa, River Cetina	39	43°31'59"N, 16°44'40"E		250	5

**Data analysis.** A list of species was compiled from all specimen data (Table 2). Comparison of species richness and assemblage composition with surrounding countries (Italy, Hungary, Serbia and Montenegro) was conducted by compiling species lists for those countries taken from the *Fauna Europaea* (Pape and Beuk 2012). Slovenia and Bosnia and Herzegovina were not included in the comparison matrix, as there are no *Dixa* species records (Pape and Beuk 2012). A species-by-country matrix was constructed and a Sørensen Index of Similarity of each pairwise comparison calculated using Primer v6 software (Clarke and Gorley 2006).

**Table 2.** Croatian *Dixa*. Species niche traits. Key: habitat type 1 = spring or eucrenal zone, 2 = stream, 3 = river, 4 = tufa barrier (barrage lake outlet); voltinism U = univoltine, B = bivoltine, M = multivoltine; occurrence Sp = Spring, Su = Summer, A = Autumn, AYR = all year round;. distribution in Europe wd = widely distributed. NA - not applicable. European Ecoregions are taken from Illies (1978); Dinaric western Balkan (5), and Pannonian lowland (11).

**Species**	**Species niche traits**				
	**Habitat type**	**Voltinism**	**Occurrence**	**Distribution**	**Ecoregion**
*Dixa dilatata* Strobl, 1900	1, 4	NA	NA	wd	5
*Dixa maculata* Meigen, 1818	1, 2, 4	U	Sp, Su, A	wd	5, 11
*Dixa nebulosa* Meigen, 1830	1, 2, 3	U, B	Sp, Su, A	wd	5, 11
*Dixa nubilipennis* Curtis, 1832	1, 4	U	Su	wd	5
*Dixa puberula* Loew, 1849	1, 2, 3, 4	U, B, M	AYR	wd	5, 11
*Dixa submaculata* Edwards, 1920	1, 2, 4	U, B, M	AYR	wd	5, 11

## Results and discussion

### List of *Dixa* species of Croatia

The following format is used for the distribution data: literature references (name of the site and in parentheses the citation of the reference and site ID); new records (life stage in which the identifications were made, i.e., adult ♂, ♀ and larvae, name of the site and in parentheses the site ID, date of collection and the collector). All the sites and their numbers are listed in Table 1.

### Genus *Dixa* Meigen, 1818

#### *Dixa dilatata* Strobl, 1900

**New records.** • 1 larva; Stream Sartuk, NP Plitvice (22); 10 Sep. 2009. •1♂; Roški Slap, Krka River, NP Krka (37); 29 Mar. 2011; M. Ivković leg.

**Remarks.** This species is newly recorded from Croatia.

#### *Dixa maculata* Meigen, 1818

**Literature references.** • tufa barrier Novakovića Brod, NP Plitvice (Ivanković et al. 2019) (24) • tufa barrier Kozjak-Milanovac, NP Plitvice (Ivanković et al. 2019) (25) • tufa barrier Labudovac, NP Plitvice (Ivanković et al. 2019) (27) • upper reach of Crna rijeka, NP Plitvice (Ivanković et al. 2019) (29) • upper reach of Bijela rijeka, NP Plitvice (Ivanković et al. 2019) (30).

**New record.** • 1 larva; Dubočanka, Papuk Mountain (14); 9 Sep. 2009.

#### *Dixa nebulosa* Meigen, 1830

**Literature references.** • River Korana in Korana village, NP Plitvice (Ivanković et al. 2019) (21) • Stream Plitvica, NP Plitvice (Ivanković et al. 2019) (23) • tufa barrier Novakovića Brod, NP Plitvice (Ivanković et al. 2019) (24) • tufa barrier Kozjak-Milanovac, NP Plitvice (Ivanković et al. 2019) (25) • tufa barrier Labudovac, NP Plitvice (Ivanković et al. 2019) (27) • upper reach of Bijela rijeka, NP Plitvice (Ivanković et al. 2019) (30) • Roški Slap, Krka River, NP Krka (Ivković et al. 2017) (37) • Skradinski buk, Krka River, NP Krka (Ivković et al. 2017) (38).

**New records.** • 1 larva; Bošćak Stream (1); 15 Apr. 2010• 1 larva; Bistrec Stream (3); 13 Jul. 2010 • 33 larvae; same site; 16 Sep. 2010 • 1 larva; same site; 18 May 2016 • 48 larvae; Kotoribski kanal (4); 18 May 2016 • 2 larvae; Plitvica, Upper Reach (5): 25 May 2009 • 10 larvae; same site; 13 Jul. 2010 • 13 larvae; Djedovica by Rupnica, Papuk Mountain (9); 9 Sep. 2009 • 1 larva; River Duboka rijeka, Papuk Mountain (12); 9 Sep. 2009 • 1♀; same site, 18.ix.2012, M. Ivković leg. • 1♀; Dubočanka, Papuk Mountain (14); 18 Sep. 2012; M. Ivković leg. • 1 larva; River Riječina, upper reach (15); 23 Sep. 2009 • 1 larva; Velika Belica (16); 24 Nov. 2009 • 1 larva; Headwater of Dobra River (17); 24 Sep. 2009 • 8♂ and 3♀; Mrežnički Brig, Mrežnica River (19); 10 Sep. 2011; M. Ivković leg. • 6 larvae; River Dretulja, Plaški (20); 9 Oct. 2009 • 3 larvae; Lake Kozjak, NP Plitvice (26); 18 Jul. 2018 • 15 larvae; same site; 16 Sep. 2016 • 4 larvae; River Kosovčica (33); 12 Jun. 2012 • 2 larvae; River Krka above the mouth of River Kosovčica (35); 19 Sep. 2012 • 2 larvae; River Krka below the mouth of River Kosovčica (36); 20 Sep. 2012 • 1♂; Roški Slap, Krka River, NP Krka (37); 29 Mar. 2011; M. Ivković leg. • 2♂; same site; 28 Apr. 2011; M. Ivković leg. • 1♂ and 1♀; Čikotina Lađa, River Cetina (39); 2005; M. Ivković leg.

#### *Dixa nubilipennis* Curtis, 1832

**Literature references.** • River Korana in Korana village, NP Plitvice (Ivanković et al. 2019) (21) • Roški Slap, Krka River, NP Krka (Ivković et al. 2017) (37).

**New record.** • 1♀; Kosovčica Spring (32); 18 Nov. 2010; M. Ivković leg.

#### *Dixa puberula* Loew, 1849

**Literature references.** • River Korana in Korana village, NP Plitvice (Ivanković et al. 2019) (21) • Stream Plitvica, NP Plitvice (Ivanković et al. 2019) (23) • tufa barrier Novakovića Brod, NP Plitvice (Ivanković et al. 2019) (24) • tufa barrier Kozjak-Milanovac, NP Plitvice (Ivanković et al. 2019) (25) • tufa barrier Labudovac, NP Plitvice (Ivanković et al. 2019) (27) • middle reach of Crna rijeka, NP Plitvice (Ivanković et al. 2019) (28) • upper reach of Crna rijeka, NP Plitvice (Ivanković et al. 2019) (29) • upper reach of Bijela rijeka, NP Plitvice (Ivanković et al. 2019) (30) • spring of Bijela rijeka stream, NP Plitvice (Ivanković et al. 2019) (31) • Roški Slap, Krka River, NP Krka (Ivković et al. 2017) (37).

**New records.** • 2♂ and 1♀; Spring Šumi, Zagorje (6); 15 Jul. 2014; M. Ivković leg. • 1♂; same site; 9 Oct. 2014; M. Ivković leg. • 1 larva; Kraljevec, Medvednica Mountain (8); 21 Jun. 2006 • 1♀; Brzaja, before N. Zvečeva, Papuk Mountain (10); 14 Jun. 2012; M. Ivković leg. • 1♀; Stream Kovačica, Papuk Mountain (11); 14 Jun. 2012; M. Ivković leg. • 3♂ and 5♀; Dubočanka, Papuk Mountain (14); 13 Jun. 2012; M. Ivković leg. • 1 larva; River Bukovačka Dobra (18); 18 Jun. 2006 • 1♂ and 1♀; Stream Plitvica, NP Plitvice (23); 28 Jun. 2007; M. Ivković leg. • 1♂; tufa barrier Novakovića Brod, NP Plitvice (24); 29 May 2007; M. Ivković leg. • 2♂ and 1♀, same site, 28 Jun. 2007; M. Ivković leg. • 1♀; tufa barrier Kozjak-Milanovac, NP Plitvice (25); 28 Jul. 2007; M. Ivković leg. • 2♂; tufa barrier Labudovac, NP Plitvice (27); 29 May 2007; M. Ivković leg. • 1♀; Roški Slap, Krka River, NP Krka (37); 17 Sep. 2010; M. Ivković leg. • 1♂; same site; 16 Dec. 2010; M. Ivković leg. • 3♀; same site; 29 Mar. 2011; M. Ivković leg. • 84♂ and 43♀; same site; 28 Apr. 2011; M. Ivković leg. • 3♂ and 6♀; same site; 29 May 2011; M. Ivković leg. • 1♂; same site; 13 Oct. 2011; M. Ivković leg.

#### *Dixa submaculata* Edwards, 1920

**Literature references.** • tufa barrier Novakovića Brod, NP Plitvice (Ivanković et al. 2019) (24) • tufa barrier Kozjak-Milanovac, NP Plitvice (Ivanković et al. 2019) (25) • middle reach of Crna rijeka, NP Plitvice (Ivanković et al. 2019) (28) • upper reach of Crna rijeka, NP Plitvice (Ivanković et al. 2019) (29) • upper reach of Bijela rijeka, NP Plitvice (Ivanković et al. 2019) (30) • spring of Bijela rijeka, NP Plitvice (Ivanković et al. 2019) (31).

**New records.** • 2 larvae; River Reka, upper reach (7); 8 Nov. 2009 • 2 larvae; Kraljevec, Medvednica Mountain (8); 21 Jun. 2006 • 1 larva; River Orašnica (34); 16 Jul. 2009 • 1 larva; River Krka above the mouth of River Kosovčica (35); 19 Sep. 2012.

### Species richness and assemblage composition

In total six species of *Dixa* (Table 2) are recorded from Croatia, collected from 39 sites (Fig. 1, Table 1). *Dixa nebulosa* is found at the greatest number of sites (25) while *Dixa dilatata* was the rarest, found only at two sites. All six species occur in the Dinaric western Balkan (Ecoregion 5), while four species occur in the Pannonian lowland (Ecoregion 11). All recorded *Dixa* species are widely distributed in Europe (Table 2). Six of the 12 recognized *Dixa* species in Europe (Pape and Beuk 2012) are now reported from Croatia. Some of the species, e.g., *Dixa puberula* and *D. nebulosa*, may eventually prove to be members of a species complex, rather than a single species (J.K. Moulton and R. Wagner, pers. comm.). Available seasonal phenological data (Table 2) revealed two species (*Dixa puberula* and *Dixa submaculata* Edwards) are multivoltine and present all year round. *Dixa maculata* and *D. nebulosa* occurred from spring through autumn. *Dixa nubilipennis* was only collected during summer.

**Figure 2.**
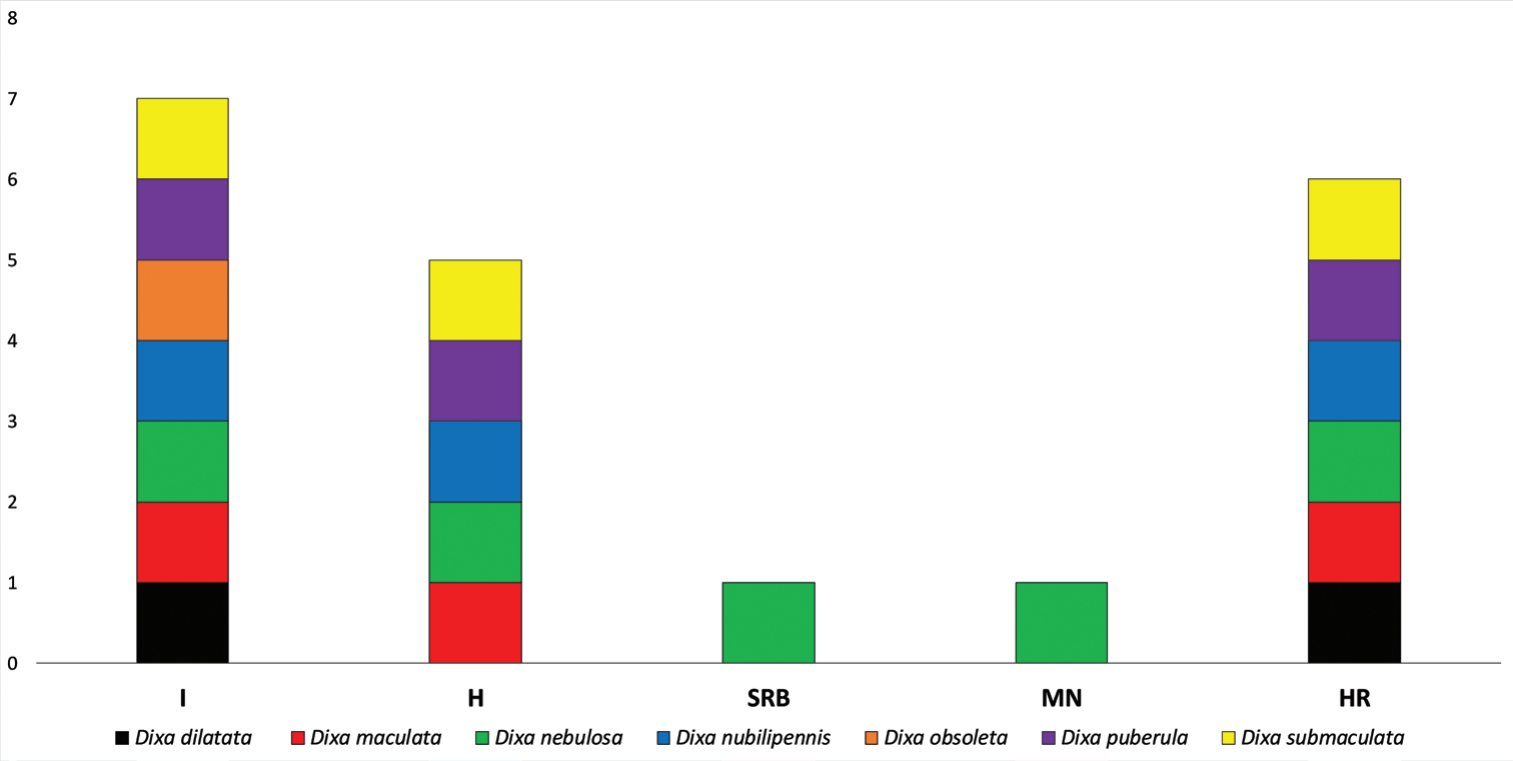
Comparison of the Croatian *Dixa* assemblage with the fauna of neighbouring countries.

Italy and Hungry have seven and five, respectively, recorded species of *Dixa*, while Serbia and Montenegro each have only a single recorded species, *Dixa nebulosa* (Fig. 2). The Sørensen Index of Similarity showed that the *Dixa* fauna of Croatia is most similar to that of Italy followed by Hungary, whereas it is least similar to that of Serbia and Montenegro (Table 3). These results were to be expected due to the low number of species recorded for Serbia and Montenegro.

**Table 3.** Sørensen Index of Similarity between *Dixa* assemblages for surrounding countries in relation to Croatia. Key: I = Italy (301 338 km²), H = Hungary (93 030 km²), SRB = Serbia (88 361 km²), MN = Montenegro (13 812 km²) HR = Croatia (56 594 km^2^).

	**I**	**H**	**SRB**	**MN**	**HR**
**I**	0				
**H**	83.33	0			
**SRB**	25	33.33	0		
**MN**	25	33.33	100	0	
**HR**	92.38	90.91	28.57	28.57	0

Comparing our list of Croatian species with published records in the *Fauna Europaea* (Pape and Beuk 2012) revealed that none of the six species treated here were previously recorded from Croatia until Ivanković et al. (2019) reported *D. maculata*, *D. nebulosa*, *D. nubulipennis*, *D. puberula*, and *D. submaculata*. Herein, we report *Dixa dilatata* as new to the dixid fauna of Croatia.

## Concluding remarks

All the recorded species have a wide European distribution and none is restricted to Croatia or to the Balkan Region. There may be a few more species of *Dixa* yet to be recorded, and, because of the high endemicity of the Dinaric area (Ivković and Plant 2015) and especially of the aquatic Diptera (Ivković et al. 2012; Pont and Ivković 2013; Kvifte et al. 2013; Kvifte and Ivković 2018), it is possible that undescribed species of *Dixa* may yet be found. In the future, collecting should be focused not only on lotic habitats but also on lentic habitats so that *Dixella* species can also be studied.
